# A case of young male with recurrent acute pancreatitis caused by an intrapancreatic gastric duplication cyst

**DOI:** 10.1007/s12328-024-01957-4

**Published:** 2024-03-27

**Authors:** Sayaka Miyamoto, Yasutaka Ishii, Masahiro Serikawa, Yumiko Tatsukawa, Shinya Nakamura, Juri Ikemoto, Yosuke Tamura, Kenichiro Uemura, Koji Arihiro, Shiro Oka

**Affiliations:** 1 Department of Gastroenterology, Graduate School of Biomedical and Health Sciences, Hiroshima University, 1-2-3 Kasumi, Minami-Ku, Hiroshima, 734-8551 Japan; 2 Department of Surgery, Graduate School of Biomedical and Health Sciences, Hiroshima University, Hiroshima, Japan; 3 Department of Anatomical Pathology, Hiroshima University Hospital, Hiroshima, Japan

**Keywords:** Gastric duplication cyst, Pancreas, Pancreatitis, Pancreatic cyst

## Abstract

Gastric duplication cyst (GDC) is a rare gastrointestinal malformation that frequently occurs in the greater curvature of the gastric antrum or corpus. Herein, we reported a case of intrapancreatic GDC found as a result of recurring pancreatitis. A 15-year-old man experienced repeated episodes of acute pancreatitis and was found to have a cystic lesion in the pancreatic tail. Contrast-enhanced computed tomography revealed a 20-mm cystic lesion with an enhanced thick wall. Endoscopic ultrasonography revealed an anechoic cyst with a three-layered wall. Magnetic resonance cholangiopancreatography and endoscopic retrograde pancreatography (ERP) revealed a connection between the cyst and the main pancreatic duct (MPD), and the duplication of the MPD. ERP showed the pancreatic duct stenosis downstream of the cyst. Although preoperative diagnosis was difficult, distal pancreatectomy was performed to prevent recurrence of pancreatitis. Pathological examination revealed that the cystic lesion was circumferentially surrounded by the pancreatic parenchyma. The epithelial lining of the cyst was crypt epithelium containing the fundic or pyloric glands and surrounded by a smooth muscle layer. The final diagnosis was intrapancreatic GDC.

## Introduction

The concept of alimentary tract duplication, as introduced by Ladd et al. [1], represents a relatively rare gastrointestinal malformation that occurs throughout the gastrointestinal tract, from the tongue to the anus. A gastric duplication cyst (GDC), which results from the disease process, has a low frequency of 4–12% [2]. Most GDCs occur in the greater curvature of the gastric antrum or corpus, and most patients are diagnosed in early childhood owing to clinical signs such as abdominal distention, the presence of abdominal masses, and vomiting [3]. In contrast, although extremely rare, GDCs may occur in the pancreas and are visualized as pancreatic cysts on computed tomography (CT) and magnetic resonance imaging (MRI) [4, 5].

Herein, we reported the case of a young male patient with intrapancreatic GDC evaluated using various imaging studies.

## Case report

**Fig. 1:**
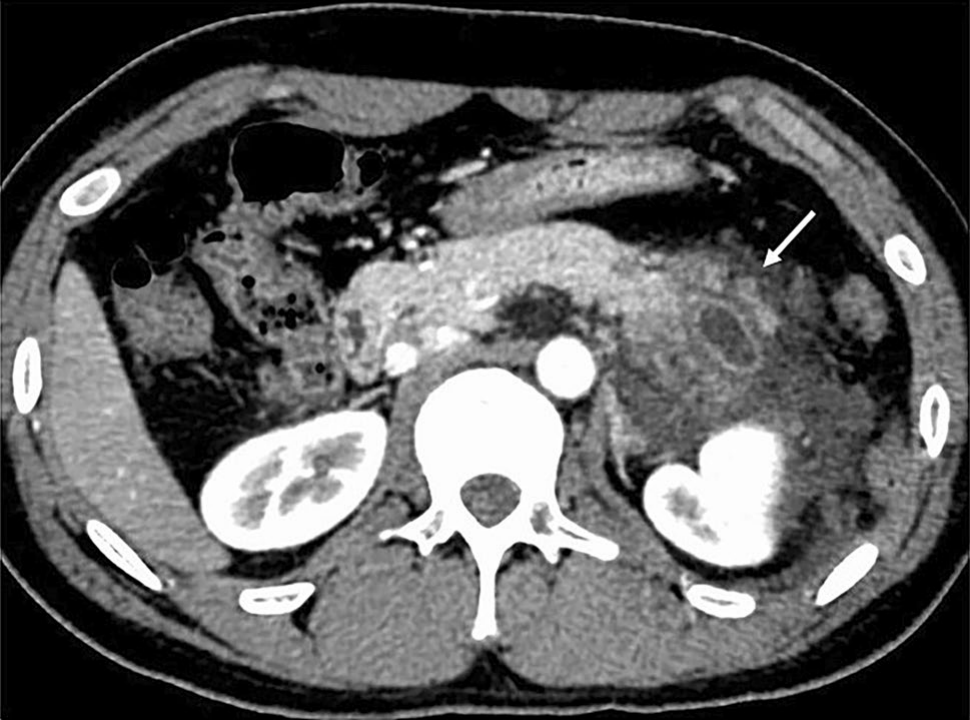
Computed tomography at the time of transfer to our hospital. Focal enlargement of the pancreatic tail, increased fat tissue density, and fluid accumulation in the surrounding area are observed (arrow). An oval cystic lesion covered with a thick capsule is observed within the swollen pancreatic tail

**Fig. 2:**
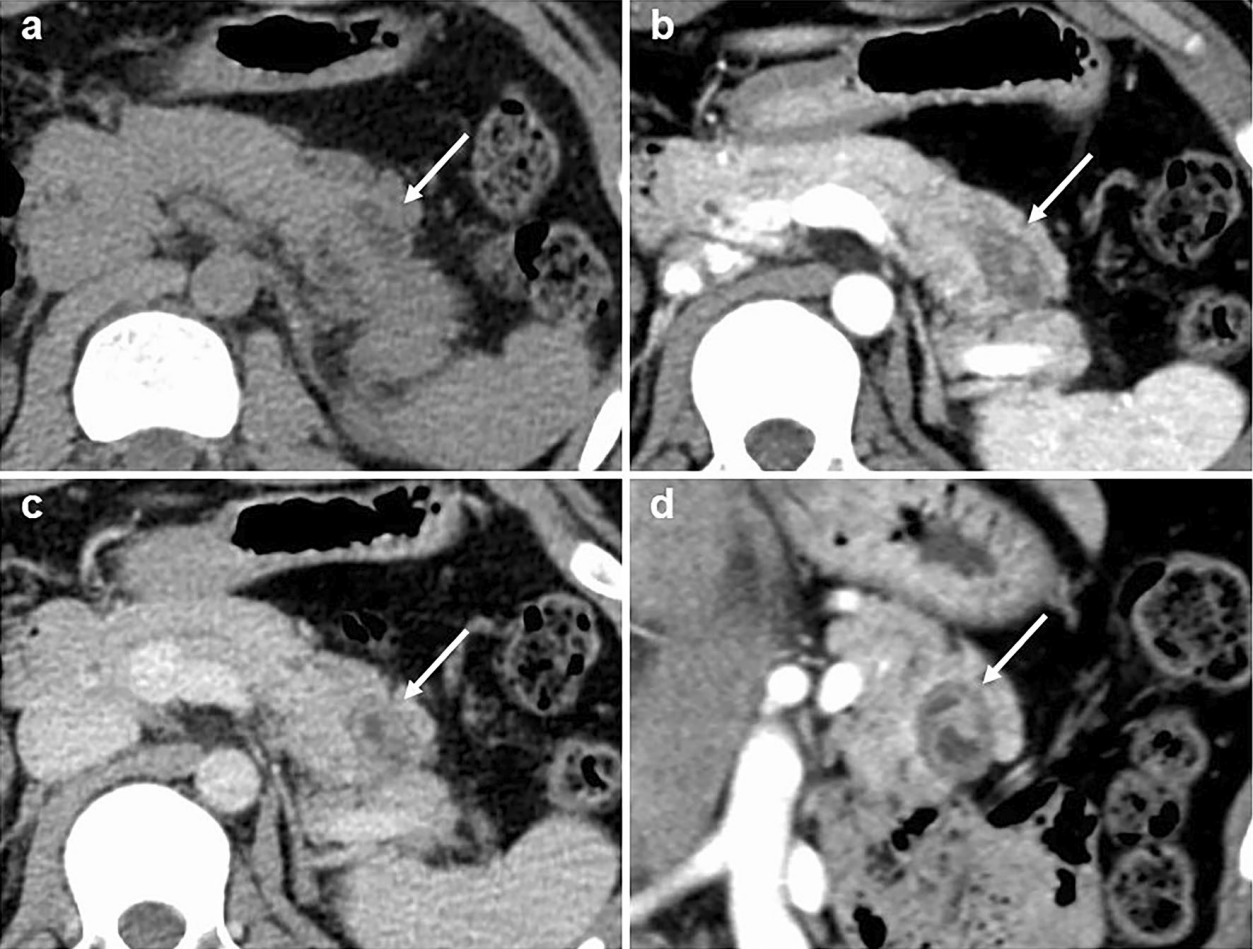
Computed tomography after remission of acute pancreatitis. A 20-mm oval cystic lesion with a thick capsule that enhances the luminal surface was observed in the pancreatic tail (arrow). (**a** plain phase, **b** arterial phase, **c** portal phase, **d** coronal portal phases)

**Fig. 3:**
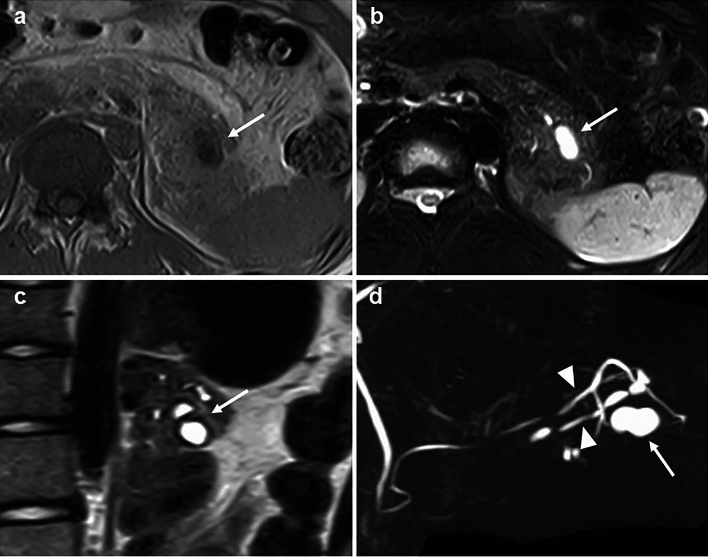
Magnetic resonance imaging. The cystic lesion (arrow) shows a hypointense signal on T1-weighted imaging (**a**) and a hyperintense signal on the T2-weighted image (**b**). The capsule shows a hypointense signal. The cyst was bilocular. (**c**). Magnetic resonance cholangiopancreatography (**d**) shows that the pancreatic tail cyst communicated with the main pancreatic duct and appeared to be two MPDs in the pancreatic tail (arrowheads)

**Fig. 4:**
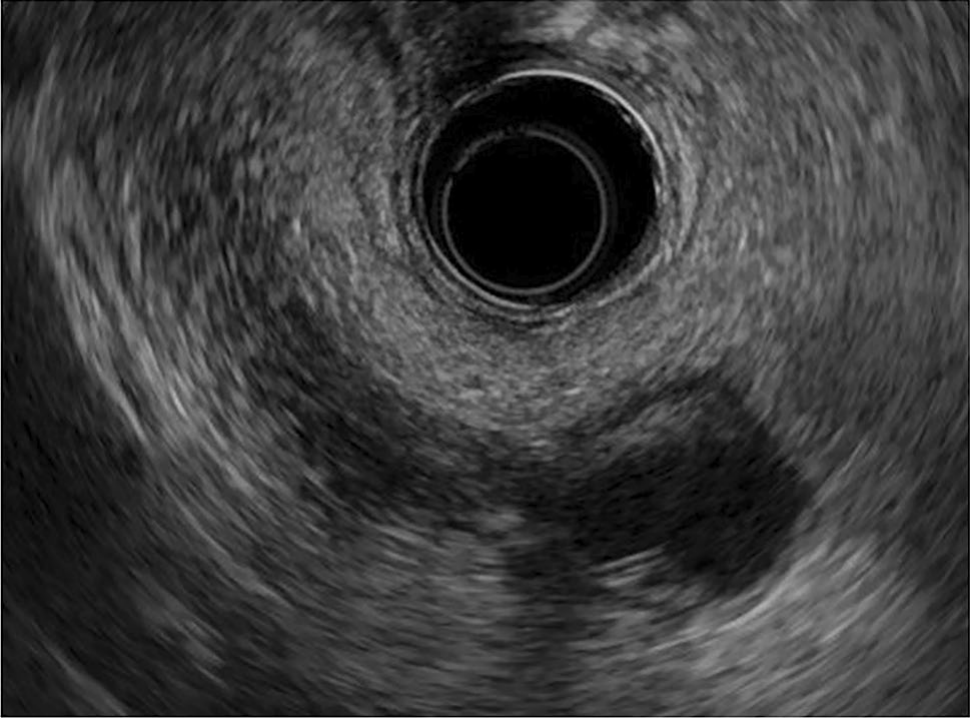
Endoscopic ultrasonography. A gourd-shaped, well-demarcated, internally anechoic cystic lesion with a three-layered wall was observed in the pancreatic tail

**Fig. 5:**
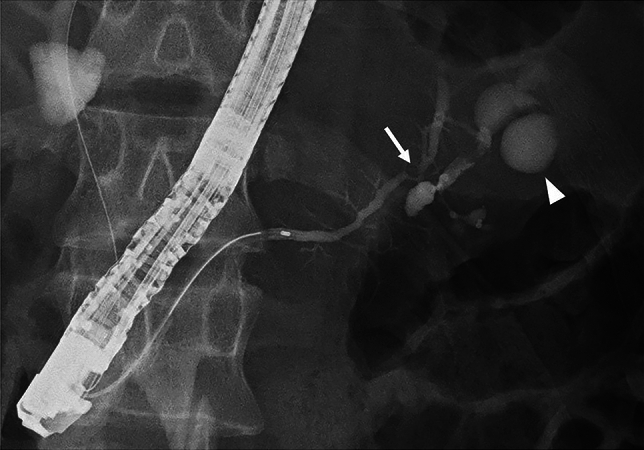
Endoscopic retrograde pancreatography. The MPD bifurcated at the pancreatic tail (arrow). Stenosis is observed at the bifurcation, and the upstream MPD is dilated and communicates with the cyst (arrowhead)

A 15-year-old boy was referred to Hiroshima University Hospital for an investigation into the underlying cause of recurrent acute pancreatitis, which had occurred three times within a 2-month period. The patient had no relevant medical or family history. The laboratory findings revealed elevated serum pancreatic enzyme levels (amylase: 1201 U/L; lipase: 3782 U/L). The tumor markers did not have elevated (carbohydrate antigen 19–9: < 2.0 U/mL; carcinoembryonic antigen: < 5.0 ng/mL). Contrast-enhanced CT revealed a focal enlargement of the pancreatic tail, increased fat tissue density, and fluid accumulation in the surrounding area (Fig. 1). In addition, an oval cystic lesion covered with a thick capsule was observed within the swollen pancreatic tail. Extrapancreatic inflammation progressed only in the left anterior pararenal space, and the unenhanced area was limited to the pancreatic tail. Based on the Japanese severity criteria [6], the patient was diagnosed with mild acute pancreatitis and recovered quickly after conservative treatment. The contrast-enhanced CT performed 3 weeks after pancreatitis remission revealed a 20-mm oval cystic lesion with a thick capsule that enhanced the luminal surface of the pancreatic tail (Fig. 2). The MRI at 3-T revealed that the cystic lesion had a hypointense signal on T1-weighted imaging and a hyperintense signal on T2-weighted imaging, and the capsule had a hypointense signal on T2-weighted imaging. The magnetic resonance cholangiopancreatography (MRCP) revealed a pancreatic tail cyst communicating with the main pancreatic duct (MPD). In addition, there appeared to be two MPDs in the pancreatic tail, suggesting MPD duplication (Fig. 3). The endoscopic ultrasonography (EUS) showed a gourd-shaped, well-demarcated, internally anechoic cystic lesion with a three-layer wall in the pancreatic tail (Fig. 4). The endoscopic retrograde pancreatography (ERP) showed the MPD bifurcated at the pancreatic tail with stenosis at its bifurcation. In addition, the upstream MPD was dilated, and communication with the cyst was observed (Fig. 5). Pancreatic juice cytology results were negative.

The cystic lesion communicated with the MPD but was unilocular and did not produce clear mucus. An intraductal papillary mucinous neoplasm was ruled out, and a retention cyst due to MPD stenosis was the primary diagnosis. Stenosis of the MPD at the pancreatic tail is considered to be the cause of recurrent acute pancreatitis. After discussing the treatment methods with the gastroenterological surgeons, spleen-preserving distal pancreatectomy was performed to prevent pancreatitis recurrence.

**Fig. 6:**
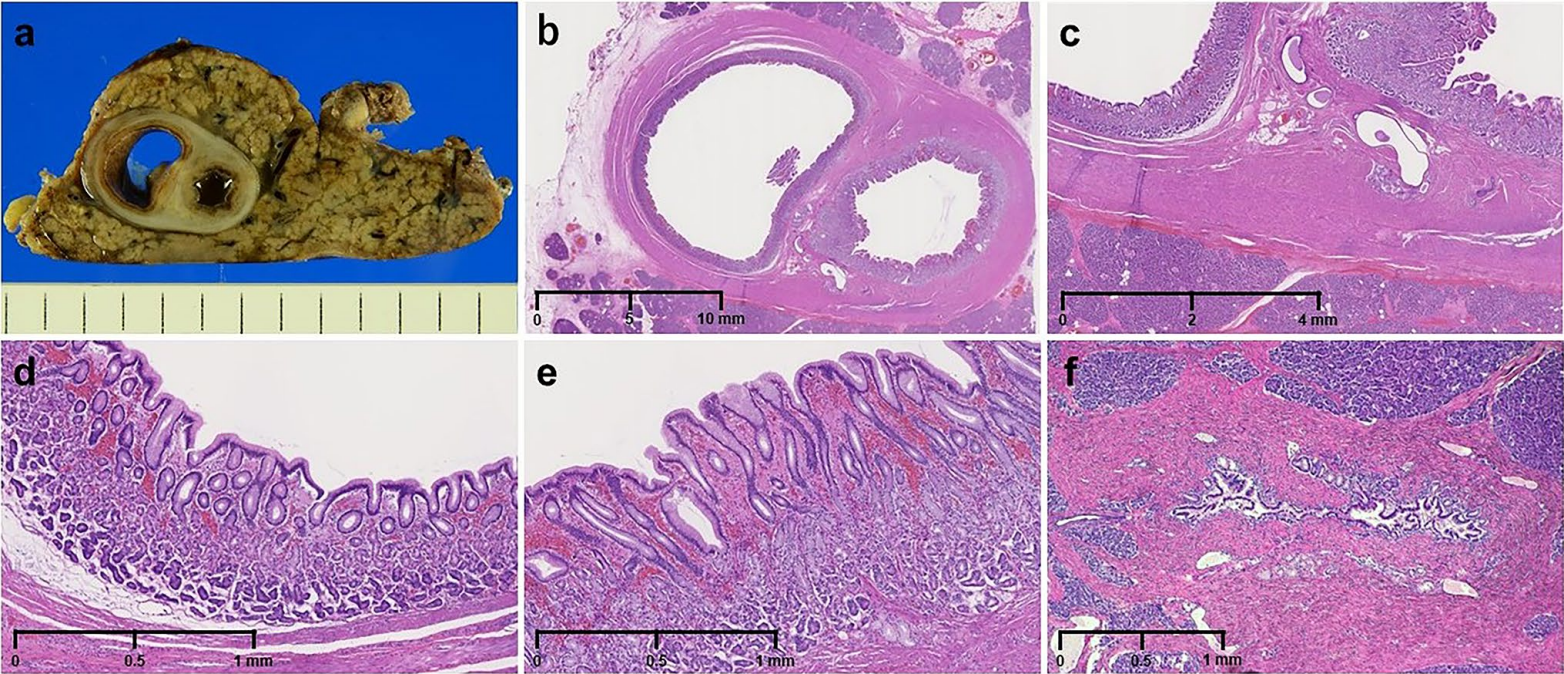
The resected specimen and histopathological findings (hematoxylin and eosin staining). A thickly encapsulated, gourd-shaped cystic lesion with well-defined boundaries is observed. The cystic lesion was circumferentially surrounded by the pancreatic parenchyma (**a**). Looped image of a cystic lesion (**b**). The outer layer is covered with smooth muscle (**c**). The mucosa is covered with an epithelium containing the fundic glands (**d**) and the pyloric glands (**e**). Inflammatory cell infiltration and fibrosis are observed around the pancreatic duct stenosis (**f**)

The resected specimen showed a 22 × 15 mm sized, thickly encapsulated, well-circumscribed cystic lesion in the pancreatic tail (Fig. 6a, b). The cystic lesion was circumferentially surrounded by the pancreatic parenchyma. The epithelial lining of the cyst was crypt epithelium containing the fundic or pyloric glands and surrounded by a smooth muscle layer (Fig. 6c, d, e). Pathologically, no continuity between the cyst and the MPD was confirmed. Inflammatory cell infiltration and fibrosis were observed around the MPD stenosis (Fig. 6f). Based on these findings, the final diagnosis of intrapancreatic GDC was established. The patient was followed-up for 3 years postoperatively without any recurrence of pancreatitis.

## Discussion

The most common location of GDCs is along the greater curvature, primarily in the antrum, and extremely rarely occurs within the pancreas, as observed in this patient. Table 1 shows the characteristics of previously reported cases [4, 5, 7], including those of this case. GDCs in two of the four cases were discovered during childhood; conversely, one patient was diagnosed at 45 years of age. Two patients complained of recurrent abdominal pain and had pancreatitis on imaging examination. In addition, MPD duplication was observed in two cases. In all these cases, GDCs significantly protruded outside the pancreas. However, our patient represents the first case where the GDC was completely surrounded by the pancreatic parenchyma, and this was confirmed both visually and histopathologically.

**Table 1:** Clinical characteristics of reported intrapancreatic GDC

Case	Age Sex	Symptoms	Location within the pancreas	Size (cm)	Imaging	Pancreatitis	MPD duplication	Treatmemt
Kamei [4]	45 years Female	Anemia	Tail	4.0	Cystic lesion	Absent	Present	DP
Lee [5]	2 years Female	Recurrent abdominal pain	Tail	2.5	Cystic lesion	Present	N/A	DP
Rao [7]	8 months Male	Hematochezia	Body–tail	30	N/A	Absent	N/A	DP
Our case	15 years Male	Recurrent abdominal pain	Tail	2.2	Cystic lesion	Present	Present	DP

*DP*, distal pancreatectomy; *GDC*, gastric duplication cyst; *MPD*, main pancreatic duct; *N/A*, not available; *PD*, pancreaticoduodenectomy

In this patient, GDC was diagnosed owing to recurrent pancreatitis. Recurrent pancreatitis frequently occurs in patients with GDCs [8, 9]. While most of the reported cases involved extrapancreatic GDCs, there was a noteworthy finding of communication between the pancreatic duct within the aberrant pancreatic lobe and the GDCs. Therefore, the suggested mechanism revolves around the obstruction of the pancreatic duct due to inflammation triggered by factors including gastric acid secretion, viscous mucus secretion, bleeding, or bile [9]. In this patient, it could not be confirmed histopathologically, but the MRCP and ERP confirmed a duplicated pancreatic duct and communication between the pancreatic duct and the GDCs. Of the three cases of intrapancreatic GDC reported to date, only one case had a pancreatic duct image evaluated; however, even in that case, communication between the duplicated pancreatic duct and GDC was observed. Considering the presence of inflammatory cell infiltration and fibrosis around the MPD stenosis, it is possible that inflammation caused by gastric acid and viscous mucus secreted from the fundic and pyloric glands and flowing into the pancreatic duct led to MPD stenosis and the development of pancreatitis. In addition, a morphological abnormality of the pancreatic duct known as MPD duplication may have contributed to pancreatic juice stasis. Therefore, in cases where intrapancreatic or peripancreatic GDC are suspected, it is necessary to evaluate the pancreatic duct images using MRCP or ERP.

Imaging findings of GDC include thick-walled cystic lesions with an enhanced inner lining on contrast-enhanced CT [10] and hypoechoic or anechoic cystic lesions with three–five layers of wall and muscular peristalsis on EUS [11–14]. In this patient, EUS did not show muscular peristalsis but showed an anechoic cystic lesion with a three-layer wall in the pancreatic tail. The multilayered structure of the cyst wall and muscular peristalsis in EUS is a finding that is not observed in pancreatic pseudocysts or pancreatic cystic neoplasms such as intraductal papillary mucinous neoplasm and mucinous cystic neoplasm and is considered to be very useful in the diagnosis of GDC. However, most cases of GDC are diagnosed during surgical resection or by pathologic examination of surgical specimens [3]. In our patient, the preoperative imaging failed to establish a GDC diagnosis. It is worth noting that GDC based on imaging findings is generally challenging, highlighting the significance of recognizing GDC as a pancreatic cystic lesion.

Owing to the challenging nature of diagnosis and the prevalence of symptomatic cases, surgical resection is often performed for GDC. According to a review by Rousek et al. [8], all symptomatic cases of GDC communication with the accessory pancreatic lobe resolve after surgery. Intrapancreatic GDC was symptomatic in all cases, including the present case, and despite its more invasive nature, pancreatectomy was considered an appropriate treatment. However, few cases of GDC that have undergone malignant transformation have also been reported [15, 16], and surgical resection may need to be considered even in asymptomatic cases [16].

Herein, we reported a rare case of a completely intrapancreatic GDC. GDCs frequently communicate with the pancreatic duct, causing pancreatitis. In addition, characteristic imaging findings may be useful in differentiating a GDC from other pancreatic cystic lesions.
